# Adaptation of Newcastle Disease Virus (NDV) in Feral Birds and their Potential Role in Interspecies Transmission

**DOI:** 10.2174/1874357901812010052

**Published:** 2018-08-31

**Authors:** Aziz-ul- Rahman, Momena Habib, Muhammad Zubair Shabbir

**Affiliations:** 1 Department of Microbiology, University of Veterinary and Animal Sciences, 54000, Lahore, Pakistan; 2 Quality Operations Laboratory, University of Veterinary and Animal Sciences, 54000, Lahore, Pakistan

**Keywords:** Avian avulavirus 1 (AAvV 1), Wild and Migratory birds, Epidemiology, Phylogeny, Newcastle Disease (ND), Fusion gene

## Abstract

**Introduction:**

Newcastle Disease (ND), caused by Avian avulavirus 1 (AAvV 1, avulaviruses), is a notifiable disease throughout the world due to the economic impact on trading restrictions and its embargoes placed in endemic regions. The feral birds including aquatic/migratory birds and other wild birds may act as natural reservoir hosts of ND Viruses (NDVs) and may play a remarkable role in the spread of the virus in environment. In addition, other 19 avulaviruses namely: AAvV 2 to 20, have been potentially recognized from feral avian species.

**Expalantion:**

Many previous studies have investigated the field prevailing NDVs to adapt a wide range of susceptible host. Still the available data is not enough to declare the potential role of feral birds in transmission of the virus to poultry and/or other avian birds. In view of the latest evidence related to incidences of AAvVs in susceptible avian species, it is increasingly important to understand the potential of viruses to transmit within the domestic poultry and other avian hosts. Genomic and phylogenomic analysis of several investigations has shown the same (RK/RQRR↓F) motif cleavage site among NDV isolates with same genotypes from domestic poultry and other wild hosts. So, the insight of this, various semi-captive/free-ranging wild avian species could play a vital role in the dissemination of the virus, which is an important consideration to control the disease outbreaks. Insufficient data on AAvV 1 transmission from wild birds to poultry and vice versa is the main constraint to understand about its molecular biology and genomic potential to cause infection in all susceptible hosts.

**Conclusion:**

The current review details the pertinent features of several historical and contemporary aspects of NDVs and the vital role of feral birds in its molecular epidemiology and ecology.

## 1 INTRODUCTION

Newcastle Disease Virus (NDV) causes wide-spread mortality in poultry but clinical and subclinical presentations have also been observed in other avian species. Its causative agent belongs to *Avulavirus* genus under *Paramyxoviridae* family [1]. This family has the varied type of members which have been responsible to cause infections in several avian species including, domestic, captive/free-ranging wild, terrestrial, and aquatic birds globally [2-6]. Genus *Avulavirus* contains all the *Avian avulaviruses* (AAvVs) specie-types, causing clinical and subclinical infection in all avian species including poultry with interspecies transmission except for avian metapneumovirus. Based on Hemagglutination Inhibition (HI) and Neuraminidase Inhibition (NI) tests [7], NDVs have been identified and classified into two different subgroups, AAvV 2 and 6 among 20 specie-types (AAvV 1 to 20) [8]. Of these, one antigenic variant of AAvV 1, causes severe infection and is responsible for epidemics in pigeon termed as Pigeon Paramyxovirus type 1 (PPMV-1) [9]. Since NDV (AAvV 1) is a highly contagious disease of poultry and other birds, it can create an alarming situation in developing countries.

So, to elucidate the interspecies transmission, attention on molecular epidemiology of AAvV 1 is the need of time. Alongside to this, the data about the molecular biology and pathogenesis of different avulaviruses 2-20 [Rahman *et al*., 2018] is not enough to evaluate the virus mutation rate. AAvV 1 (or ND) is an OIE (Office International Epizootics; world organization for animal health) notifiable avian infection implicated with significant economic losses as well as natural genetic depletion of diverse hosts [5]. In the above context, the intention of this review is to provide a summary of overall pertinent evidence related to geographical distribution and susceptible host density of AAvV 1 with its temporal, terrestrial, and host density. However, up to date and comprehensive overview of the genetic diversity is the main consideration to assess the evolutionary analysis of NDVs among avian species. This review summarises, compares and discusses the available literature on NDVs genetic diversity and may be helpful to investigate the mutable aspects of NDVs in all genes particularly fusion (*F*) gene.

### 1.1 Geographical Distribution

**Fig. (1):**
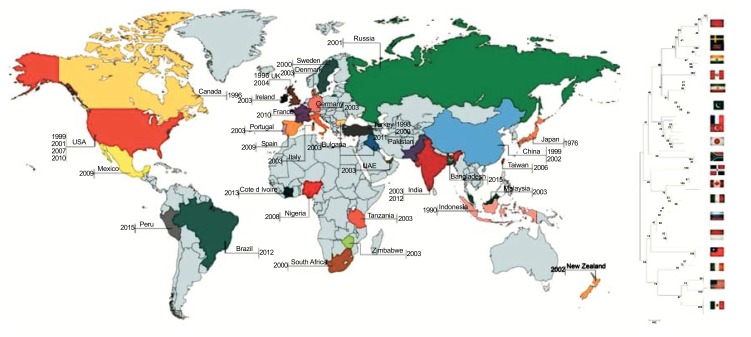
NDV from wild bird origin isolated in different regions/countries, also showed the geographical evolutionary relationship among these countries where it is endemic. For example, NDV from Pakistan, Iran, China and India grouped in same cluster.

Several avian species can spread the numerous microbes across the globe, which are harmful to captive and free-ranging birds including poultry [10, 11]. During the last few years, NDV and influenza virus have been found to be transmitted by migratory birds across the globe [12, 13]. It provided the strong evidence that wild avian species have been implicated in the transferring of NDV to domestic and wild bird population as biological carriers/ natural reservoir [14]. ND was first described in 1926 in Newcastle-on-Tyne, England (from where it got its name) and on the island of Java, now part of Indonesia, although there have been some suggestions that there may have been earlier outbreaks. It appears that initially the disease spreads rapidly in Asia [15] but slowly reported a series of devastating outbreaks around the globe including Africa, Central America and parts of South America [16-18]. A lot of outbreaks in different avian species associated with virulent NDV were reported from North America (USA, Canada and Costa Rica), Europe (France, Italy, England, Scotland, Spain and Russia), Africa (Kenya) and Asia (China, Japan, India, Israel, Saudi Arabia and Kazakhstan) [19] (Fig. **1**).

Alongside poultry, NDV has been isolated from wild birds with incidences of virus transmission from wild psittacine/ cormorants to chickens in the United State during the 1970s and 1990s [20, 21]. The findings from these studies warrant the consistent monitoring of wild birds in a pause of virus dissemination. In India, many bird’s species including chicken, turkey, quail, emu, duck, peacock, pigeon, duck and guinea fowl have been found as potential susceptible host for NDV infection [22-29]. The current NDV epidemics in feral birds in Asian countries especially neighbours of India like Pakistan [17, 30] and Bangladesh [31] pose the threats to disease dissemination by sharing countries boundaries and the movement of migratory birds from one to another country. Regards to epidemiological context, different NDV strains are circulating in the field to infect the domestic and wild birds [32] that highlight the importance of the characterization of current prevailing NDV isolates from indigenous captive/semi-captive bird and from clinical or subclinical infection in free-ranging feral birds [33]. However, the interspecies transmission propensity of field circulating viruses may raise concerns about the efficacy of vaccine, used in the field. Various outbreaks have revealed the existence of vaccine strains of domestic poultry in wild birds from Mexico [34]. Moreover, vaccine NDV strain was confirmed in Australian wild bird [35] including feral birds in Japan [36], Finland [37], China [38], America [39] and in central Nigeria [40].

Except for Asian countries, zoo birds in Israel and Mexico have become infected with virulent NDV strains similar to those causing infection in domestic birds, which indicated the spill-over of virus in the environment [41]. In this account, the similar findings have been observed in Africa [42] and other continents [43, 44] which raised the concerns regarding the potential role of wild birds in shedding of NDVs in environment and transmission to poultry. Moreover, the existence of anti-NDV antibodies in feral birds of South Africa [45], Burkina Faso [46], and Nigeria [47] has also exposed the susceptibility of a wide range of avian hosts. The molecular detection, evolutionary dynamics and viral genetic manipulation will be helpful to control the epidemics and thus, we need to focus on the molecular and geographically epidemiology of NDV strains and should be perceived as an epidemiological alert to re-assess the control and quarantine measures against NDV [48, 49].

### 1.2 Genetic Variability of Newcastle Disease Virus

Avian avulaviruses are RNA viruses, having non-segmented, a single-stranded negative-sense genome with helical capsid symmetry. It has a molecular weight of about 5 × 106 Da, with approximately 15.2 kb in length and encodes six structural and two non-structural proteins in the order 3'-NP–V/W/P-M-F–HN-L-'5 [50]. The surface projections on envelope are approximately 8 nm long, present on the HN molecule, whilst F molecules form smaller projections. The F protein has significance as a type-I integral membrane protein with the trans-membrane domain located in the carboxyl-terminal region followed by a short cytoplasmic domain [51], responsible for virulence. During the infection, the first HN protein of virus attaches to a cell and then fusion protein makes linkage for the invasion of viral genetic material into the host cell [52]. The virulence of NDV depends on the primary molecular determinant which is known as F protein cleavage site having specific amino acid sequence pattern and position [53]. The susceptible host got an infection when the cellular proteases cleave an inactive F0 precursor protein into F1 and F2 subunits [51]. The NDV pathotype totally depends on the F protein cleavage site having dibasic amino acids in velogenic and mesogenic strains, while the F protein of lentogenic NDV isolates lack this motif [51, 54]. The NDV isolates from migratory birds have shared the similar specific dibasic amino acid at the cleavage site of F gene which has been detected in chickens from the same area during an outbreak [55]. Usually, the virulent strain has RQK/RRF residue pattern whereas K/RQG/ERL residues [53] have been observed at specific position 112-117 in the NDV strain of low virulence [56] (Table **1**). In comparison, a mutative NDV isolate has also been detected from a dove having a K for Q substitution at residue 114; evidence of adaptation of NDV to the environment [57, 58].

**Fig. (2):**
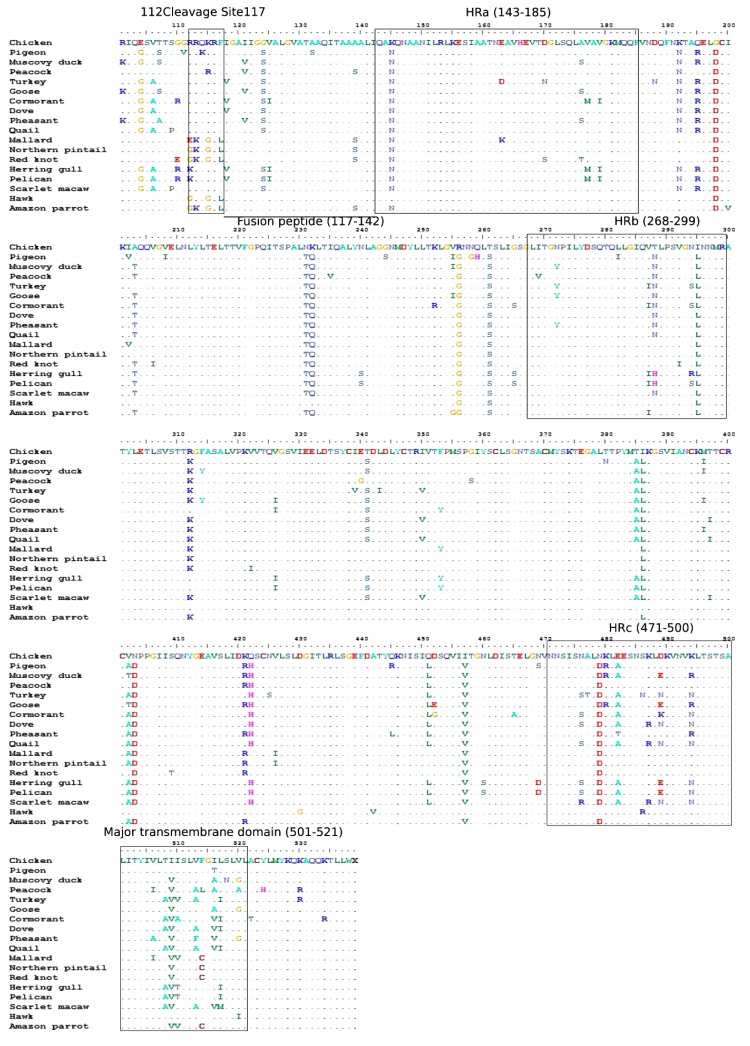
Alignment of deduced amino acid sequence of complete F gene of NDV isolate from different wild birds with reference of chicken isolate with accession numbers AY562991, JX901111, FJ872531, HQ011508, JN942045, HG326605, GQ288385, GQ429293, JX854452, KC808512, KJ920203, EF612277, EF564816, JN255779, JN941993, KC808510, KC808489, KC808493 from chicken to amazon parrot, respectively. Structurally and functionally important residues are boxed and highlighted based on residue profile to explore similarities among them.

Although, F protein is highly conserved among all isolates consisting of a series of Heptad Repeat (HR) regions [51], some sort of mutations have also been observed in it such as the mutation of the L at position 154 in the HR1 region from residues 130 to 170 interferes in cell fusion [59]. In addition, substitutions in the other conserved regions like HR2 region from N-terminal to the TM domain, a helical region HR3 domain from residues 263 to 289 and another helical domain HR4 from residues 81 to 102 [60, 61] have been observed (Fig. **2**). Based on the evolutionary estimation, the *F* gene divergences of 1-18% between NDVs isolates from chicken-origin and wild-origin [62], and it was highly similar to the hawk origin NDV strain with 2% divergence. Although, NDVs from wild bird origin had nucleotide homologies of up to 99.8% and low homologies of 82% with chicken origin NDVs [63].

In addition, NDVs originated from quail and dove showed a minor nucleotide difference (15%) when compared to isolates from chicken, but these did not exhibit any significant antigenic differences (1%) between them. Similarly, duck and goose shared only 1% nucleotide dissimilates (Table **2**). To date, the complete genome sequences of a number of strains of AAvV 1-20 [64, 65] have been published. The genetic information was available only from Nigeria [66], Pakistan [17, 67], India [29, 33, 68, 69], China [70] and South Africa [71], but still, a wide range of birds remains uncovered to investigate the potential of NDV to transmit and infect diverse host range.

### 1.3 Host Range

According to the world organization for animal health (WHO), ND is a contagious disease affecting more than 250 avian species around the globe [72]. In this context, a lot of free-ranging aquatic fowls are considered as a potential carrier of AAvV 1 [7, 73]. In 1946, after the first evidence of virulent strain of NDV in United States of America and Mexico, wild migratory avian and white storks were found to be susceptible for virulent NDV strain [74, 75]. Such kind of circumstantial evidence revealed the potential of virus and mode of transmission AAvV 1 by implicating wild birds to poultry [14]. It is not only about migratory birds, some recreational birds and captive wild birds also seemed to be as a natural reservoir/ carrier and susceptible hosts of NDVs [76]. Previously, the incidences of NDVs in wild and migratory aquatic birds [37], pigeons [36, 40], Peacock (17, 29, 33, 68], pheasant [67], duck and geese [70] have been reported.

NDV as a mutable RNA virus has the ability to cross interspecies barrier by mutating itself. Related evidence has been observed in Luxembourg, where lentogenic viruses highly similar to the LaSota strain were isolated from waterfowl subsequent to spill over and interspecies adaptation [42]. In another previous study related to Turkey, the causative virus was identified in genotype Ia along with several cases of NDVs in wild birds which were kept in live bird’s market [77]. Taken together, it was suggested that viruses from wild birds may spill over into poultry. Based on AAvVs genetic diversity analysis, the viral transmission between wild birds and poultry was proved, previously [13, 42, 78, 79]. Furthermore, the NDV strains isolated from wild birds could cause outbreaks in chickens. For example, NDV isolates from migrating cormorants were identified as the likely source of epidemic in poultry. The interaction between wild birds and poultry happens frequently; the wild birds possibly play a pivotal role in the evolution of NDV for the adaptation of environment [78, 80].

Interestingly, it has been shown that double-crested cormorant is an important reservoir of NDVs among all feral bird population [81]. Similarly, pigeons and mallards have been suggested to be the reservoir of NDVs [82, 83]. In New Zealand, NDV has been isolated from a red-breasted musk parrot following its seizure after illegal importation forms Fiji [84]. Specifically, waterfowls are considered important reservoirs of NDVs and may act as a carrier for NDV transfer to poultry, causing outbreaks of different pathogenicity [12, 85, 86]. In recent years, a disease resembling those of ND has been reported in ducks and geese in the regions of China [87]. Thus, it could be inferred that feral birds are carriers of virulent strains but transmission pattern of virulent NDV strains is not yet fully understood.

Small wild birds mainly Passeriformes can also transmit NDVs due to their peri-domestic habits and some avian species are playing a significant role in epizootiology of NDVs [4]. NDV had also been recovered from domestic duck farms [88] and wild birds [89], which raise concerns regarding NDV transmission among several avian species. So, few reports on mortality in free-living birds other than feral pigeons (*Columba livia),* recently captured teal (*Anas crecca*) [90], double-crested cormorants (*Phalacrocorax auritus*), white pelicans (*Pelecanus erythrorhynchos*), and gulls (*Larus spp.)*, raised the concerns about apparent epizootic nature of the virus [91]. In North America, during ND outbreaks, virulent strains of NDV of non-poultry origin have been reported from cormorants, gulls and pelicans [62, 92], and NDV strain of low virulence has been isolated from gulls, shorebirds, and waterfowl as part of avian influenza surveillance [12, 93]. NDV strains of variable virulence have also been isolated from wild pigeons and doves [94, 95]. In view of host susceptibility, NDVs have been reported from a quite wide range of various feral birds, including wild, aquatic and recreational birds (Table **1**). Although a variety of wild birds are susceptible to ND, the information available is limited due to the non-availability of clinical/necropsy samples and poor disease surveillance in feral avian population. Thus, there is a need for NDV surveillance in all susceptible wild birds to insight the evolution and adaptation of virus for controlling ND outbreaks worldwide.

### 1.4 Clinico-pathological Presentation of NDV

Based on pathogenicity, NDVs are classified into different four stains [19]. First, the Velogenic Viscerotropic NDV (VVNDV) formerly Dolye form, responsible for acute and lethal infections causing hemorrhagic lesions in visceral organs. Second, the Velogenic neurotrophic NDV (VNNDV) formerly Beach form, responsible for high mortality involve in respiratory and neurologic signs (gut lesions are absent). Third, the Mesogenic NDV formerly Beaudette form causes low mortality with acute respiratory disease and nervous signs. At four, the Lentogenic or asymptomatic enteric NDV formerly Hitchner form is a virulent virus that appears to replicate primarily in the gut with mild or inn-apparent respiratory infections [96].

Wild and migratory birds were considered the natural reservoirs of NDV and harboured mainly Lentogenic strains but occasionally Velogenic strains have potential to shed in the environment [42, 89, 97]. Mortality in wild birds such as pigeon, juvenile double-crested cormorants (*Phalacrocorax auritus*) and teal (*Anas crecca*) has also been reported due to the infection caused by Velogenic strains [25, 29, 62, 74, 92]. Highly virulent viruses from psittacines and in a variety of zoo and live market birds have also been isolated [42, 98-100]. Depend on infection, strain, dose rate, route of exposure, hostage, immunological status and environmental conditions, signs can range from no clinical presentation to neurologic signs, paralysis, and/or acute death [19]. However, few pathotypes are responsible for peracute infection with almost 100% mortality to subclinical disease with no lesions [93].

#### 1.4.1 Galliformes, Anseriformes and Columbiformes Birds

Turkey is a susceptible host to vNDV with clinical signs of depression, nasal discharge, blood-tinged diarrhea and nervous incoordination [101]. Partridges and pheasants are considered to be extremely sensitive to vNDV [102] with similar clinical signs as observed in chickens from the acute onset with high mortality, severe nervous signs to inapparent infection [7]. In a study, neurological signs in experimentally infected ducks with a mesogenic NDV strain were also observed [103]. Similarly, the goose was also reported as a susceptible host for NDV [104, 105] with moderate to severe depression, anorexia, diarrhea, ocular and nasal discharges, and swelling of the eyelids [87]. Histopathologically, the susceptibility of birds was characterized by multifocal areas of ulceration and hemorrhages in the esophagus, gizzard, and multifocal necrosis of the intestinal mucosa [106]. Ulceration and fibrin deposition in the intestinal mucosa and over the cecal tonsils, severe atrophy of lymphoid organs, and lymphoid depletion, multifocal areas of necrosis in the pancreas and less frequently in the liver were also observed. In a few cases, the brain was affected by neuronal degeneration present [106]. These types of investigation have clear evidence about the interspecies transmission prosperity of NDV.

In pigeons, ND is caused by pigeon specific variant of ND virus known as pigeon paramyxovirus-1 (PPMV-1) [58]. The outbreak in pigeon was reported first time in the Middle East during 1970 and spread to Europe during 1980 and now become endemic around the globe [81]. Neurological signs and diarrhea are major clinical signs seen mainly in young birds [107]. Gross lesions in pigeons infected with PPMV-1 from natural outbreaks consist of pancreatic necrosis, enteritis, and proventricular hemorrhages [108]. Histologically, lesions consist of non-suppurative encephalitis, multifocal necrosis in spleen, bursa, liver, larynx, and pancreas, and multifocal accumulation of lymphocytes in several organs [108] with spleen enlargement and perivascular cuffing in the cerebellum and brainstem [101].

#### 1.4.2 Psittaciformes, Passeriformes and Suliformes Birds

NDVs were also isolated from non-domesticated species of *Psittaciformes* and *Passeriformes* [4] with neurological signs in psittacine and *Passeriformes* birds [109]. Different cases of ND were also reported in six different states of the United States in 1991 [110]. Clinical signs included tremors, lateral recumbency, respiratory distress, greenish diarrhea, ruffled plumage, and head drawn back between the shoulders, and eventual death. The VVND viruses were isolated from affected birds, including yellow-headed Amazon parrots (*Amazona ochrocephala oratrix*), yellow-naped Amazon parrots (*Amazona ochrocephala auropalliata*), cockatiels (*Nymphicus hollandicus*), and Canaries (*Serinus canarius*). In another study, VVNDV was isolated from experimentally infected birds including budgerigars (*Melopsittacus undulates*), Amazon parrots (*Amazona ochrocephala auropalliata)*, and Canaries (*Serinus canaries)* with neurological signs consisting of tremors, ataxia, wing droop, and uni or bilateral leg paralysis [111].

Histopathology following exposure to VVNDV results in haemorrhages and necrosis of the intestinal mucosa, haemorrhages on the skullcap and around the orbit, fibrinous peritonitis, hepatosplenomegaly, focal hepatic necrosis, airsacculitis, and hemorrhagic tracheitis in the Canaries, Amazon parrots, and budgerigars. These birds were also able to spread the virus and infect cage-mates. Shedding of VVNDV has been observed for more than 1 year in Amazon parrots and for more than 80 days in budgerigars, both enabled in the spread of virus in the environment [111]. NDV outbreaks in double-crested cormorant (*Phalacrocorax auritus*) populations [112] were also reported with neurological signs including prominent gross lesions of enlarged and mottled spleen associated with bursal atrophy and multifocal hemorrhagic foci in the meninges [112]. Histologically, lesions having multifocal nonsuppurative encephalitis with areas of gliosis appeared more prominent in the cerebellar white matter, interstitial nephritis and multifocal myocarditis [113]. Isolation of NDVs from commercial or feral birds reported no associated clinical signs [114, 115]. So, based on previous studies, existence and transmission of NDVs among different avian species including domesticated and wild birds with a variable clinical infection impose the continuous surveillance of wild and migratory birds.

### 1.5 Phylogenetic Analysis of Wild Originated NDVs

Two major classes (class I and II) of NDVs were premeditated according to genome length-based phylogenetic analysis [74]. In class I, there are atleast nine different genotypes, while in class II comprised of eighteen genotypes (I–XVIII) (Maminiaina *et al*., 2010; Meng *et al*., 2012). In class II, the genotypes VI and VII were further divided into nine (a-i) and five (a-e) sub-genotypes, respectively [116]. Noteworthy, Munir *et al*. [17] concluded that the isolates can be divided into six broadly distinct lineages (1 to 6). Of these, lineage 3 and 4 further subdivided into four (a-d) and five (a-e) sub-lineages, respectively. According to the phylogenetic analysis, most of NDV strains from pigeon or dove were classified into genotype VI [117] often considered as mesogenic strains, and a little information about other genotypes NDV from *Columbidae* was recorded [32, 92]. The diversity was found in amino acid sequences of *F* genes of NDVs, all publicly available isolates at GenBank^®^, indicating the evolutionary and mutative potential of NDVs. Dissimilarities among vaccine strain and field isolates from genotype V of class II have also been reported [74, 118], that aid in viral shedding, further, the persistence of NDVs in backyard poultry and free-living wild birds, explaining why vNDV caused sporadic outbreaks in the poultry industry until recently [119].

Similarly, phylogenetic relationships of non-virulent NDV strains in shorebirds and waterfowl provide support for the spread of viruses among different avian species and geography (12, 94). Virulent NDVs of identical genetic make-up were isolated from wild pigeons and doves in the United States, providing the evidence of virus spread from the different origin within different avian species and geography of North America [95, 96]. However, intercontinental transport of viruses may be attributed to the trade of racing pigeons. Thus, dissemination of NDVs within North America is supported, but evidence for intercontinental virus spread by migratory birds is limited. Thus, previous studies on genetic diversity among strains of NDVs revealed that some strains from wild birds were phylogenetically related with NDVs isolated from live-bird markets (12, 42). One previous study from Africa revealed the different host adaptation of NDVs where, the domestic pigeons originated NDVs of genotype VI have been found responsible for causing infection in different birds, such as feral pigeons and doves [57]. Such type of transmission has also been reported with high mortality rates in other studies [96, 120] This actually points out the viral evolution for adaptation of hosts leads to constrain in its eradication.

So far, among wild birds, virulent NDVs seem enzootic not only in cormorants in North America [62] but also in pigeons worldwide [87, 95]. Additionally, viruses from genotype XVIII have been reported from wild birds in the United States. Phylogenetically, the viruses originated from wild birds showed a close relationship to strains isolated from live birds markets [121], raise the questions about interspecies transmission of NDVs for the conservation of endangered wild birds. Such findings highlight the potential of NDVs to transmit *via* migration of wild birds, which are natural reservoirs of NDVs [55]. Furthermore, isolation of virulent NDVs from wild aquatic birds raise the concern that wild and migratory waterfowls could play a role as a long-distance vector of virulent NDV, highlighting the need for increased NDV surveillance in aquatic fowls [89].

**Fig. (3):**
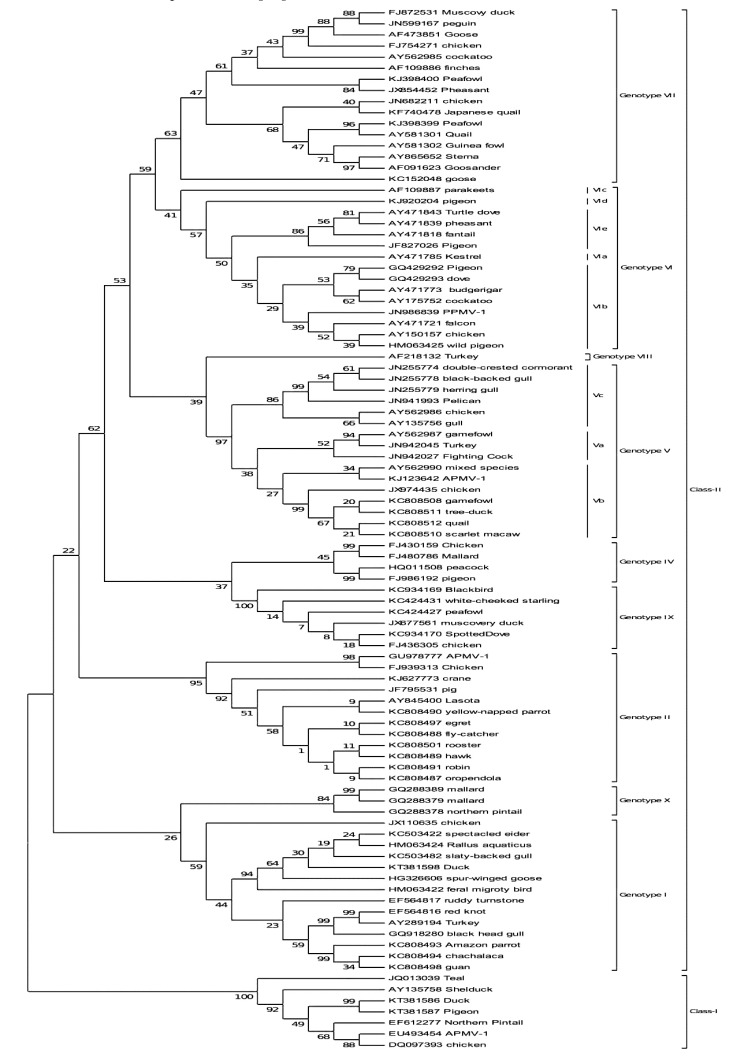
Molecular phylogenetic analysis of fusion (F) gene by a maximum likelihood method conducted in MEGA6. All NDV isolates showed an evolutionary relationship with chicken origin NDVs and also with other wild or captive bird's origin NDVS.

To estimate the evolutionary distances between wild birds originated NDV strains, complete *F* gene-based phylogenetic analysis was performed by the maximum composite likelihood method in MEGA6 software [122]. The evolutionary analysis of already reported wild bird originated NDVs revealed the strong relationship among all NDVs originated from wild birds, domestic birds and poultry (Fig. **3**).

In this figure, NDVs from different feral birds showed a strong evolutionary relationship with the poultry originated strains as mentioned in class I and II and also clustered within different genotypes. Generally, the velogenic NDV strains from poultry origin have been clustered in genotype VII but NDVs from wild bird’s origin are also found in genotype IX, V and class I beyond its pathotype properties. Such type of grouping of NDV strain in different genotype points out the continuous evolution of field circulating viruses, which may be due to the vaccine failure because, in many countries, NDV epidemics have been observed in vaccinated birds. Therefore, the active surveillance and monitoring of wild birds can help us prevent the disease transmission in all susceptible host and evolution of NDVs.

## CONCLUDING REMARKS

Based on the previous molecular investigations, it can be concluded that poultry and non-poultry host cohabiting raise NDV epidemics around the globe particularly in endemic countries. Phylogenetic analysis of wild and domestic originated NDVs provided such evidence, which supports that wild birds are contributing to the global redistribution of NDVs alongside the regular vaccination but only in poultry. The cleavage site in F protein of the NDVs has been found to be an important factor contributing to the pathogenesis in hosts. However, few mutations have been observed in different isolates originated from different bird’s species, result in variability of virulence. So, the investigation of genetic variation and isolate’s evolutionary analysis can provide valuable data on characterization, epidemiology among different avian species and diagnosis of NDVs circulating in the environment. In future, the attention of the global scientific community towards these aspects would help to elucidate the complete epidemiology trends of NDVs and to validate the robustness of diagnostic screening, particularly in endemic countries. In addition, a continuous surveillance of wild and migratory/aquatic fowls will share the information to design the appropriate control strategies.

## Figures and Tables

**Table 1:** NDV susceptible host range with geographical distribution.

**Accession No.**	**Host**	**Scientific Names**	**Year**	**Country**	**Genotype**	CSP*	**Virulence**	**References**
FJ436303	Chicken	*Gallus gallus domesticus*	1986	China	IX	RRQRRF	virulence	[123]
FJ872531	Muscovy duck	*Cairina moschata*	2002	China	VII	RRQKRF	virulence	[124]
GQ288389	Mallard	*Anas platyrhynchos*	1999	USA	IIa	EKQGRL	avirulence	[48]
AY865652	Sterna	*S. hirundo*	2001	Russia	Vb	RRQRRF	virulence	[125]
JQ013039	Teal	*Anas crecca*	2010	France	Class I	ERQERL	avirulence	[126]
KJ627773	Crane	*Gruidae*	1992	India	-	GKQGRL	avirulence	[127]
KJ398400	Peafowl	*Pavo cristatus*	2012	India	VII	RRQRRF	virulence	[127]
AY581302	Guinea fowl	*Numididae*	2000	India	-	RRQRRF	virulence	[128]
GQ288391	Mottled duck	*Anas fulvigula*	2001	USA	IIa	GKQGRL	avirulence	[48]
GQ288378	Northern pintail	*Anas acuta*	1987	USA	IIa	GKQGRL	avirulence	[48]
EF564816	Red knot	*Calidris canutus*	2001	USA	Ia	GKQGRL	avirulence	[13]
AY289194	Turkey	*Meleagris gallopavo*	2003	Canada	-	GKQGRL	avirulence	[129]
JX854452	Pheasant	*Phasianus colchicus*	2011	Pakistan	VII	RRQKRF	virulence	[67]
KC934169	Blackbird	*Turdus merula*	2008	China	IX	RRQRRF	virulence	[130]
KC934170	Spotted dove	*Spilopelia chinensis*	2008	China	IX	RRQRRF	virulence	[130]
KC424431	White-cheeked starling	*Sturnus cineraceus*	2008	China	IX	RRQRRF	virulence	[131]
JN255774	Double-crested cormorant	*Phalacrocorax auritus*	2010	USA	VII	KRQKRF	virulence	[62]
JN255778	Black-backed gull	*Larus marinus*	2010	USA	VII	KRQKRF	virulence	[62]
JN255779	Herring gull	*Larus argentatus*	2010	USA	VII	KRQKRF	virulence	[62]
JN941993	Pelican	*Pelecanus*	2008	USA	-	KRQKRF	virulence	[132]
KC808510	Scarlet macaw	*Ara macao*	2009	Mexico	Vb	RRQKRF	virulence	[34]
KC503422	Spectacled eider	*Somateria fischeri*	2007	USA	Ib	GKQGRL	avirulence	[133]
KC503482	Slaty-backed gull	*Larus schistisagus*	2007	USA	Class I	GKQGRL	avirulence	[133]
AY562985	Cockatoo	*Cacatuidae*	1990	Indonesia	VIId	RRQKRF	virulence	[134]
KF740478	Japanese quail	*Coturnix japonica*	2003	India	VII	RRQKRF	virulence	[135]
JN599167	Penguin	*Spheniscidae*	1999	China	VII	RRQKRF	virulence	[136]
AY471773	Budgerigar	*Melopsittacus undulatus*	1995	Turkey	VIId	RRQKRF	virulence	[137]
HG326606	Spur-winged goose	*Plectropterus gambensis*	2008	Nigeria	I	GKQGRL	avirulence	[42]
HM063424	Water rail	*Rallus aquaticus*	*2005*	China	I	GKQGRL	avirulence	[138]
HM063425	Wild pigeon	*Columba livia*	2003	China	VI	RRQKRF	virulence	[138]
AF109886	Finches	*Fringillidae*	1997	UK	Ve	RRQKRF	virulence	[139]
AF091623	Goosander	*Mergus merganser*	1996	UK	Vb	RRQRRF	virulence	[139]
AY471818	Fantail	*Rhipidura*	1996	Turkey	VIIc	GRQKRF	avirulence	[137]
AY471721	Falcon	*Falconiforme*	2000	Turkey	VIIf	RRQKRF	virulence	[137]
AY471785	Kestrel	*Falco tinnunculus*	1999	Turkey	VIId	RRQKRF	virulence	[137]
AY471843	Turtle dove	*Streptopelia turtur*	1996	Turkey	VIIb	GRQKRF	avirulence	[137]
KC808497	Egret	*Ardea alba*	2009	Mexico	II	GRQGRL	avirulence	[34]
KC808491	Robin	*Turdus grayi*	2009	Mexico	II	GRQGRL	avirulence	[34]
KC808490	Yellow-napped parrot	*Amazona auropalliata*	2009	Mexico	II	GRQGRL	avirulence	[34]
KC808489	Hawk	*Buteo brachyurus*	2009	Mexico	II	GRQGRL	avirulence	[34]
KC808487	Oropendola	*Zacua spp.*	2009	Mexico	II	GRQGRL	avirulence	[34]
KC808493	Amazon parrot	*Amazona autumnalis*	2009	Mexico	Ia	GRQGRL	avirulence	[34]
KC808494	Chachalaca	*Ortalis vetula*	2009	Mexico	Ia	GRQGRL	avirulence	[34]
KC808498	Guan	*Penelopina nigra*	2009	Mexico	Ia	GRQGRL	avirulence	[34]
FJ938175	Sparrow	*Passer domesticus*	2005–2007	China	VII	RRQKRF	virulence	[140]
EF564817	Ruddy turnstone	*Arenaria interpres*	2002	USA	Ia	GKQGRL	avirulence	[13]
JX855036	Crested Ibis	*Nipponia nippon*	2013	China	VIId	RRQKRF	virulence	116
JF820295	Ostrich	*Struthio camelus*	2011	Iran	VII	RRQKRF	virulence	141

**Table 2:** The nucleotide based genome homology of different NDVs isolated from a wide range of wild/feral birds

	**% Nucleotide Divergence and Homology Between Each Pair of Isolates**																		
	**Chicken**	**Pigeon**	**Muscovy** **duck**	**Peacock**	**Turkey**	**Goose**	**Cormorant**	**Dove**	**Pheasant**	**Quail**	**Mallard**	**Sterna**	**Northern** **pintail**	**Red** **knot**	**Herring** **gull**	**Pelican**	**Scarlet** **macaw**	**Hawk**	**Amazon** **parrot**
**Chicken**		0.01	0.01	0.01	0.01	0.01	0.01	0.01	0.01	0.01	0.01	0.01	0.01	0.01	0.01	0.01	0.01	0.00	0.01
**Pigeon**	0.17		0.01	0.01	0.01	0.01	0.01	0.01	0.01	0.01	0.01	0.01	0.01	0.01	0.01	0.01	0.01	0.01	0.01
**Muscovy** **duck**	0.17	0.11		0.01	0.01	0.00	0.01	0.01	0.01	0.01	0.01	0.01	0.01	0.01	0.01	0.01	0.01	0.01	0.01
**Peacock**	0.14	0.14	0.13		0.01	0.01	0.01	0.01	0.01	0.01	0.01	0.01	0.01	0.01	0.01	0.01	0.01	0.01	0.01
**Turkey**	0.18	0.15	0.14	0.15		0.01	0.01	0.01	0.01	0.01	0.01	0.01	0.01	0.01	0.01	0.01	0.01	0.01	0.01
**Goose**	0.17	0.12	0.01	0.14	0.15		0.01	0.01	0.01	0.01	0.01	0.01	0.01	0.01	0.01	0.01	0.01	0.01	0.01
**Cormorant**	0.18	0.15	0.13	0.16	0.10	0.14		0.01	0.01	0.01	0.01	0.01	0.01	0.01	0.00	0.00	0.01	0.01	0.01
**Dove**	0.15	0.12	0.11	0.12	0.06	0.11	0.07		0.01	0.00	0.01	0.01	0.01	0.01	0.01	0.01	0.00	0.01	0.01
**Pheasant**	0.18	0.13	0.08	0.15	0.15	0.08	0.16	0.13		0.01	0.01	0.01	0.01	0.01	0.01	0.01	0.01	0.01	0.01
**Quail**	0.15	0.13	0.11	0.13	0.07	0.11	0.08	0.01	0.14		0.01	0.01	0.01	0.01	0.01	0.01	0.00	0.01	0.01
**Mallard**	0.11	0.16	0.16	0.13	0.18	0.16	0.18	0.15	0.17	0.16		0.01	0.01	0.01	0.01	0.01	0.01	0.01	0.01
**Sterna**	0.18	0.12	0.09	0.14	0.14	0.09	0.15	0.12	0.11	0.12	0.17		0.01	0.01	0.01	0.01	0.01	0.01	0.01
**Northern** **pintail**	0.10	0.16	0.15	0.14	0.17	0.15	0.17	0.15	0.16	0.15	0.04	0.16		0.01	0.01	0.01	0.01	0.01	0.01
**Red** **knot**	0.14	0.17	0.16	0.15	0.17	0.17	0.19	0.16	0.17	0.16	0.12	0.16	0.12		0.01	0.01	0.01	0.01	0.01
**Herring** **gull**	0.18	0.15	0.14	0.16	0.11	0.14	0.04	0.08	0.16	0.09	0.18	0.15	0.18	0.19		0.00	0.01	0.01	0.01
**Pelican**	0.18	0.15	0.14	0.16	0.10	0.14	0.04	0.08	0.16	0.08	0.18	0.15	0.17	0.18	0.01		0.01	0.01	0.01
**Scarlet** **macaw**	0.16	0.13	0.11	0.13	0.07	0.12	0.08	0.02	0.14	0.01	0.16	0.13	0.16	0.17	0.09	0.08		0.01	0.01
**Hawk**	0.02	0.17	0.17	0.14	0.18	0.17	0.19	0.15	0.18	0.15	0.11	0.17	0.10	0.14	0.18	0.18	0.16		0.01
**Amazon** **parrot**	0.11	0.15	0.14	0.13	0.16	0.15	0.17	0.13	0.14	0.14	0.10	0.14	0.09	0.08	0.17	0.16	0.15	0.11	
